# Stability Evaluation of Extemporaneously Compounded Vancomycin Ophthalmic Drops: Effect of Solvents and Storage Conditions

**DOI:** 10.3390/pharmaceutics13020289

**Published:** 2021-02-23

**Authors:** Christopher Ross, Basir Syed, Joanna Pak, Vishal Jhanji, Jason Yamaki, Ajay Sharma

**Affiliations:** 1 Chapman University School of Pharmacy, Chapman University Irvine, Room 267, 9401 Jeronimo Road, Irvine, CA 92618, USA; chross@chapman.edu (C.R.); bsyed@chapman.edu (B.S.); jpak@chapman.edu (J.P.); yamaki@chapman.edu (J.Y.); 2 Department of Ophthalmology School of Medicine, University of Pittsburgh School of Medicine, Pittsburgh, PA 15213, USA; jhanjiv@upmc.edu

**Keywords:** MRSA, keratitis, vancomycin, compounding, stability

## Abstract

Vancomycin is the drug of choice for methicillin-resistant *Staphylococcus aureus* keratitis and other ocular infections. Vancomycin ophthalmic drops are not commercially available and require compounding. The present study was designed to investigate the stability of vancomycin ophthalmic drops in normal saline, phosphate-buffered saline (PBS), and balanced salt solution (BSS) while stored at room temperature or under refrigeration. Vancomycin ophthalmic drops (50 mg/mL) were aseptically prepared from commercially available intravenous powder using PBS, BSS, and saline. Solutions were stored at room temperature and in a refrigerator for 28 days. The vancomycin stability was tested by a microbiology assay and high-performance liquid chromatography HPLC analysis immediately after formulation and at days 7, 14, and 28 after storage at room temperature or under refrigeration. The pH, turbidity was also tested. Vancomycin formulations in PBS, BSS and normal saline had initial pH of 5; 5.5; 3 respectively. The formulation in PBS developed turbidity and a slight decrease in pH upon storage. Microbiological assay did not show any change in zone of inhibition with any of the formulation upon storage either at room temperature or under refrigeration. HPLC analysis did not detect any decrease in vancomycin concentration or the accumulation of degraded products in any of the formulations upon storage either at room temperature or under refrigeration. Vancomycin ophthalmic drops prepared using PBS, BSS, and normal saline were stable up to the tested time point of 28 days, irrespective of their storage temperature.

## 1. Introduction

Keratitis is an infection of the cornea caused by bacteria, fungi, herpes virus, or acanthamoeba. It is a sight-threatening medical emergency requiring prompt and effective intervention. *Staphylococcus aureus* is the second most common pathogen causing bacterial keratitis, although the exact incidence of infection varies by geographic location [1,2]. Methicillin-resistant S. aureus (MRSA) are strains of S. aureus resistant to the all antibiotics within the β-lactam class, except ceftaroline [3,4,5]. The acquisition of the mecA or mecC genes, which lead to alternative penicillin-binding proteins, account for the most common mechanism of resistance that results in the methicillin-resistant phenotype [6,7,8,9]. Although historically considered a hospital-acquired infection, MRSA clones are increasingly being encountered among community infections [10,11,12]. MRSA is known to cause a variety of ocular infections, including keratitis [13,14,15]. The incidence of MRSA ocular infections in the United States has been rising in recent years, with multiple studies reporting that MRSA accounts for roughly 30–40% of all S. aureus cases of keratitis [16,17,18].

The American Academy of Ophthalmology recommends 1.2%, 2.5%, or 5% vancomycin ophthalmic drops for keratitis caused by MRSA [19]. Alternatively, fluoroquinolone ophthalmic drops, which are commercially available, have been anecdotally used for MRSA keratitis, but ocular MRSA infections respond poorly, and the incidence of fluoroquinolone resistance remains a serious concern [20,21]. On the contrary, MRSA-associated keratitis isolates are largely responsive to vancomycin, which is the gold standard MRSA treatment for a wide range of different infection types, and resistance is rare [21,22]. A recent study has reported that 5% vancomycin ophthalmic drops have a higher efficacy in treating MRSA keratitis compared to lower concentrations [22]. However, vancomycin ophthalmic drops are not available commercially and require preparation by a licensed compounding pharmacy. The compounding pharmacy is required to be a member of the Pharmacy Compounding Accreditation Board 152 and designated by the FDA as a 503A and/or 503B facility. The American Academy of Ophthalmology’s preferred practices pattern guidelines recommend the use of sterile normal saline for compounding vancomycin and its subsequent storage under refrigeration [19]. However, case reports and circumstantial evidence show that, besides normal saline, phosphate-buffered saline (PBS) and balanced salt solution (BSS) have also been used for compounding vancomycin. The requirement of compounding vancomycin ophthalmic drops leads to an increased financial cost for the patient, and the drops are typically recommended to be discarded after seven days despite the possibility of a longer shelf-life. Additionally, some patients with keratoprosthetics will require the use of ophthalmic vancomycin for longer durations [23,24]. These clinical needs necessitate the evaluation of the stability of compounded vancomycin ophthalmic drops. There are no comprehensive studies that evaluate the stability of compounded vancomycin ophthalmic drops in normal saline, PBS, and BSS with storage conditions either under refrigeration or at room temperature. Therefore, the present study was designed to investigate the stability of compounded vancomycin ophthalmic drops in normal saline, PBS, and BSS while stored at room temperature or under refrigeration, using the United States Pharmacopeia-recommended microbiological assay [25] and a high-performance liquid chromatography (HPLC) analysis. 

## 2. Materials and Methods

### 2.1. Preparation of Vancomycin Ophthalmic Drops

Vancomycin eye drops were prepared aseptically from commercially available lyophilized powder of vancomycin hydrochloride (Mylan Pharmaceuticals, Morgantown, WV, USA) intended for intravenous use. A 50-mg/mL solution of vancomycin hydrochloride was prepared using three different vehicles: 1× phosphate-buffered saline (PBS), balanced salt solution (BSS), and normal saline. Each solution was then aliquoted into two equal parts with storage at room temperature and at 4 °C in a refrigerator.

The formulations were tested for pH and turbidity immediately after formulation and then at days 7, 14, and 28 after storage.

### 2.2. Stability Testing of Vancomycin Ophthalmic Drops

The stability of vancomycin ophthalmic drops prepared in three different vehicles and stored under two different conditions were tested using a HPLC chemical analysis and microbiological assay. The assays were performed using freshly prepared solutions immediately after formulation and then at days 7, 14, and 28 after the preparation and storage.

(a) Chemical Analysis of vancomycin using HPLC method

The HPLC method was used to quantify the vancomycin concentrations in the aliquots of the three ophthalmic formulations stored under two different conditions immediately after formulation and then at days 7, 14, and 28 after preparation and storage. The HPLC chromatograms were also run to screen for any additional peaks indicative of degradation products. A freshly prepared solution of vancomycin was used as a standard. The vancomycin ophthalmic formulation samples were diluted to be analyzed at 1-mg/mL concentrations. The sample concentration was calculated against five-point (2, 1, 0.5, 0.25, and 0.125 mg/mL) vancomycin standard curve concentrations. The HPLC analysis was done on a Hitachi LaChrome Ultra,(Hitachi High-Tech, Dallas, TX, USA) equipped with an autosampler and UV/Vis detector, using Vydac 218tp54, 250 × 4.6 mm, 5-um particle size, and C18 column. Mobile phase A, 0.1% trifluoroacetic acid (TFA) in water, was prepared by mixing 1 mL of TFA to 1 L of water and sonicating for 10 min. Mobile phase B, 0.1% TFA in acetonitrile, was prepared by mixing 1 mL of TFA to 1 L of acetonitrile and sonicating for 10 min. The flow rate was 1.0 mL/min with the following gradient method: hold at 10% mobile phase B+ 90% mobile phase A for 4 min, 10–35% mobile phase B + 65% mobile phase A for 7 min, and hold at 10% mobile phase B + 90% mobile phase A for 4 min. A 20-uL sample injection volume of the samples was used, and detection was performed at the 280-nm wavelength.

(b) Microbiological Assay

The United States Pharmacopeia method was used for the microbiological assay of vancomycin [25]. The media containing peptone (6 g/L), yeast extract (3 g/L), beef extract (1.5 g/L), agar (15 g/L), and distilled water (1 L) was used to prepare agar plates. Eight-millimeter holes were punched in the inoculated agar plates. Aliquots were obtained from each of the three ophthalmic formulations stored under two different conditions immediately after formulation and then at days 7, 14, and 28 after preparation and storage. These aliquots were diluted to an obtained a 1-μg/50-μL concentration of vancomycin, and 50 μL of each of these ophthalmic formulation samples was added to the punched hole in the agar plate. A freshly prepared solution of 1-μg/50-μL vancomycin was used as a standard. The plates were then incubated for 24 h at 37 °C, and the zone of inhibition in mm was noted down. The formulations were prepared twice, and each of the samples was analyzed in duplicate. The presented data is a replicate of *n* = 4.

### 2.3. Statistical Analysis

The data are presented as mean ± standard error of mean. Statistical analysis was performed using GraphPad Prism software (GraphPad Prism, Version 8, San Diego, CA, USA). The data were analyzed using one-way ANOVA, followed by Dunnett’s post-hoc test. A *p*-value of <0.05 was considered statistically significant.

## 3. Results

### 3.1. Appearance and PH of Vancomycin Ophthalmic Formulations

Vancomycin formulated in PBS and stored at room temperature or under refrigeration remained clear for 14 days after preparation, but subsequently, it developed visible turbidity under both the storage conditions. The BSS and normal saline formulations stored at room temperature or under refrigeration remained visibly clear up to the tested time point of 28 days.

Table 1 shows the pH of the vancomycin formulations immediately after preparation and then at various days after storage at room temperature or under refrigeration.

Vancomycin formulated in PBS, BSS, and normal saline had an initial pH of 5, 5.5, and 3, respectively. The pH of the PBS formulation decreased to 4.5 after seven days in both the samples stored at room temperature and under refrigeration. After this initial decrease, no further decrease in pH was noted in this PBS formulation up to the tested time point of 28 days. On the other hand, the BSS and normal saline formulations did not show any change in pH with storage either at room temperature or under refrigeration.

### 3.2. HPLC Quantification of Vancomycin Ophthalmic Formulations

**Figure 1:**
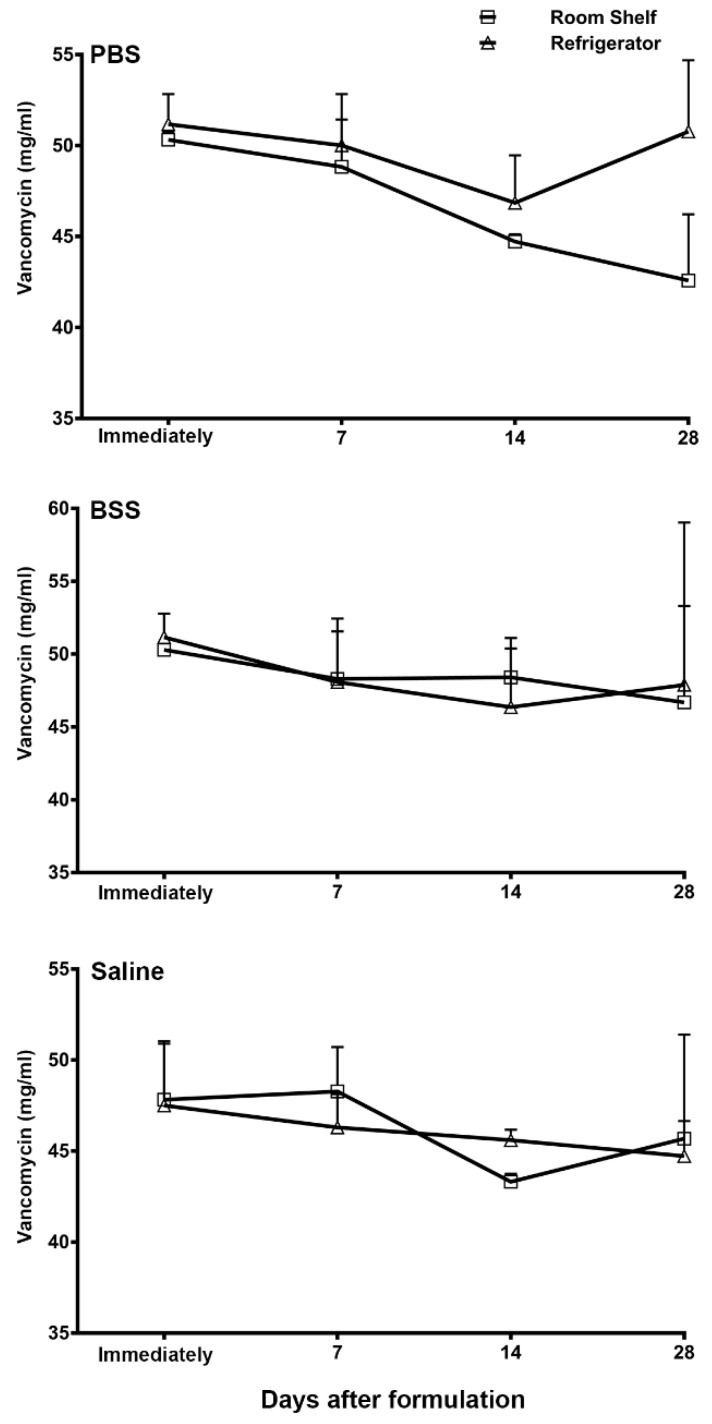
Quantification of vancomycin (mg/mL) in the phosphate-buffered saline (PBS), balances salt solution (BSS), and normal saline formulations analyzed by high-performance liquid chromatography (HPLC) immediately after formulation and then at days 7, 14, and 28 after storage at room temperature or under refrigeration. The data is presented as mean ± standard error of mean (SEM).

Figure 1 shows the HPLC quantification of the PBS, BSS, and normal saline formulations of vancomycin immediately after preparation and then at various days after storage at room temperature or under refrigeration. The HPLC analysis did not detect any significant decrease in the vancomycin concentration after storage in any of these formulations. Furthermore, the stability of vancomycin was not affected by the storage temperature, since no significant difference in the vancomycin concentrations was noted whether the formulations were stored at room temperature or under refrigeration.

The HPLC chromatograms showed a vancomycin peak with an average elution time of 9.5 min in all these formulations when analyzed immediately after preparation.

Subsequently, an identical vancomycin peak with a similar elution time was also detected in these formulation samples upon analysis. There were no additional peaks in the HPLC chromatogram of the vancomycin formulations upon storage either at room temperature or under refrigeration compared to the freshly prepared vancomycin standard, suggesting that vancomycin did not have any degradation product formations upon storage in any of the three formulations stored either at room temperature or under refrigeration.

### 3.3. Microbiology Assay of Vancomycin Ophthalmic Formulations

**Figure 2:**
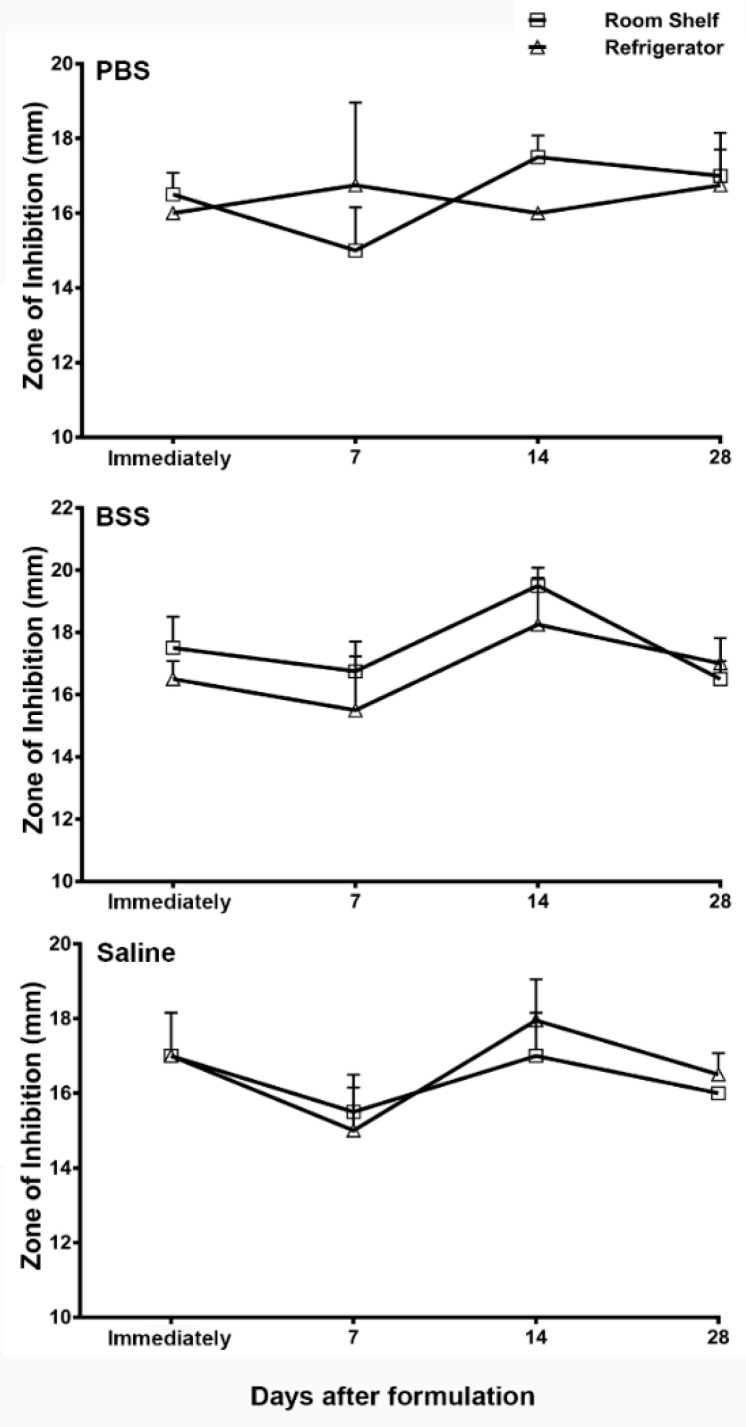
Zone of inhibition (in mm) of the PBS, BSS, and normal saline vancomycin formulations analyzed by a microbiology assay immediately after formulation and then at days 7, 14, and28 after storage at room temperature or under refrigeration. The data is presented as mean ± standard error of mean (SEM).

A change in the bactericidal activity of antibiotics in microbiology assays is a reliable and activity-based indicator of a loss of potency. Therefore, we next tested the potency of vancomycin in the ophthalmic formulations using the microbiological assay method recommended by the United States Pharmacopeia [25]. Figure 2 shows the zone of inhibition of vancomycin in the formulations prepared using PBS, BSS, and normal saline. The zone of inhibition assay was performed in these formulations immediately after preparation, and then at 7 days, 14 days, and 28 days after compounding. No significant decrease in the zone of inhibition was observed in any of the formulations tested at the various time points upon storage when compared to the zone of inhibition analyzed in these formulations immediately upon preparation. Therefore, the microbiological assay data suggest that the vancomycin formulations did not lose potency upon storage either at room temperature or under refrigeration. 

## 4. Discussion

The topical application of vancomycin is the sight-saving treatment for keratitis caused by vancomycin-susceptible bacteria resistant to other antibiotic classes [20,21,22,26,27,28]. Topical vancomycin has also been used for preventing devastating ocular infections in patients who receive keratoprosthetics [23,24,29]. The treatment duration for keratitis typically requires approximately one to two weeks of application, and keratoprosthetics prophylaxis can last for a much longer duration [20,21,22,23,24,26,27,28,29]. There are currently no commercially available vancomycin ophthalmic formulations, thus necessitating compounding by a licensed pharmacy. Vancomycin ophthalmic drops need to be compounded under sterile conditions. These requirements make vancomycin ophthalmic drops expensive. The repeated compounding of vancomycin drops for the duration of a treatment further adds to the cost and the inconvenience of making additional trips to the pharmacy for picking up the drops, which can adversely affect patient adherence. The results of the present study demonstrate that compounded vancomycin eye drops are stable up to the tested time points of 28 days. Based on our data, it is feasible to compound larger quantities of vancomycin ophthalmic drops and then aliquot them into multiple smaller sterile containers for dispensing to the patient. This approach will reduce the cost while still maintaining the sterility if the patient is adequately counseled on opening and using one container at a time.

The American Academy of Ophthalmology’s preferred practice pattern guidelines recommend the use of sterile normal saline for compounding vancomycin [19]. However, a variety of other solvents have also been used. The differences in ionic composition, pH, and buffering capacity of these solvents can affect the physicochemical stability of vancomycin. In the present study, we used a microbiological assay, as well as a HPLC-mediated chemical analysis, to compare the stability of vancomycin in three different formulations. Microbiological testing is a functional assay providing direct evidence of bactericidal activity. If an antibiotic has conformational changes, it may cause a decrease in its potency without causing any detectable changes in the HPLC analysis. On the other hand, a slight chemical degradation may not affect the antimicrobial activity of an antibiotic in the microbiology assay, but the accumulation of degradation products could be a concern for potential toxicity. The HPLC analysis is a sensitive assay to detect any additional peaks arising from the degrading products. Using these two assays, the results of the present study demonstrate that the stability of vancomycin is not affected by the type of solvent, since the formulations in the three different solvents did not show any significant decrease or dissimilarity in stability over time. Additionally, no extra peaks of impurities were detected in any of the formulations by the HPLC analysis, suggesting that vancomycin does not undergo degradation in these solvents.

Further, we tested whether the storage temperature can affect the stability of vancomycin for any of these ophthalmic formulations. The results of the present study demonstrated no significant difference in vancomycin stability in any of the three formulations based on their storage condition at room temperature or under refrigeration. Formulations or drugs that require refrigeration impose many practical challenges, such as their transportation from the pharmacy to a patient’s home and the storage while the patient is at work or is traveling. Based on the results of the present study, it may be recommended that patients can carry and store the compounded ophthalmic drops at room temperature, avoiding excessive heat or direct sunlight as is generally recommended. Our results suggest that there may not be a need for refrigeration over the concerns of losing the potency while storing vancomycin ophthalmic drops at room temperature. Vancomycin is commonly used in the antimicrobial therapy to treat hospital-acquired MRSA, and the common route of administration is a slow intravenous infusion in 5% dextrose or normal saline. The stability of vancomycin at room temperature during a slow intravenous infusion is a concern. Therefore, several previous studies have tested the stability of vancomycin for a shorter duration, ranging from 1 h, 48 h, and 17 days when diluted in 5% dextrose or saline at ambient temperature [30,31,32,33,34,35]. In agreement with the results of the present study, these studies also demonstrated that vancomycin is stable when stored at room temperature. The results of the present study extend that stability for a longer duration of 28 days. Further, the data from these previous studies also showed that plastic tubing or iv bags do not cause vancomycin adsorption [30,31,32,33,34,35].

Although our study did not detect any differences in the stability of vancomycin over 28 days of storage either at room temperature or refrigeration, we did observe a difference in the pH and turbidity. It is worthwhile to note that vancomycin formulated in PBS developed a visible turbidity, with a slight decrease in pH in storage both at room temperature and under refrigeration. Vancomycin is available as a hydrochloride salt, and its dissolution results in an acidic solution. As anticipated, the formulation in normal saline offered no buffering, and this formulation had the most acidic pH. On the other hand, both BSS and PBS offered some buffering capacity, and the pH of the vancomycin formulations in these solvents were significantly less acidic. Acidic ophthalmic formulations may cause a stinging sensation upon instillation, and our results suggest that vancomycin formulated in normal saline may be less comfortable to the patient compared to the formulations in PBS and BSS.

## 5. Conclusions

In conclusion, the results of the present study demonstrate that vancomycin ophthalmic drops prepared using PBS, BSS, and normal saline are stable up to the tested time point of 28 days irrespective of their storage at room temperature or under refrigeration.

## Figures and Tables

**Table 1:** The pH of vancomycin formulations in phosphate-buffered saline (PBS), balanced salt solution (BSS), and normal saline immediately after formulation and then at days 7, 14, and 28 after storage at room temperature or under refrigeration. RT: room temperate.

Solvent	Day 0	Day 7	Day 14	Day 28
PBS—RT	5.0 ± 0	4.5 ± 0	4.5 ± 0	4.5 ± 0
PBS—4 °C	5.0 ± 0	4.5 ± 0	4.5 ± 0	4.5 ± 0
BSS—RT	5.5 ± 0	5.5 ± 0	5.5 ± 0	5.5 ± 0
BSS—4 °C	5.5 ± 0	5.5 ± 0	5.5 ± 0	5.5 ± 0
Saline—RT	3.0 ± 0	3.0 ± 0	3.0 ± 0	3.0 ± 0
Saline—4 °C	3.0 ± 0	3.0 ± 0	3.0 ± 0	3.0 ± 0
